# Divergence in cellular markers observed in single-cell transcriptomics datasets between cultured primary trabecular meshwork cells and tissues

**DOI:** 10.1038/s41597-025-04528-5

**Published:** 2025-02-14

**Authors:** Alice Tian, Sangbae Kim, Hasna Baidouri, Jin Li, Xuesen Cheng, Janice Vranka, Yumei Li, Rui Chen, VijayKrishna Raghunathan

**Affiliations:** 1 Department of Molecular and Human Genetics, Baylor College of Medicine, Houston, Texas 77030 USA; 2 University of Houston, College of Optomtery, Houston, TX 77204 USA; 3 Center for Translational Vision Research, Gavin Herbert Eye Institute, Department of Opthalmology, University of California Irvine School of Medicine, Irvine, CA 92617 USA; 4 Oregon Health & Sciences University, Portland, OR USA; 5 Biomedical Research, Novartis, Cambridge, MA 02139 USA

**Keywords:** Data integration, Transcriptomics

## Abstract

The trabecular meshwork within the outflow apparatus is critical in maintaining intraocular pressure homeostasis. *In vitro* studies employing primary cell cultures of the human trabecular meshwork (hTM) have conventionally served as surrogates for investigating the pathobiology of TM dysfunction. Despite its abundant use, translation of outcomes from *in vitro* studies to *ex vivo* and/or *in vivo* studies remains a challenge. Given the cell heterogeneity, performing single-cell RNA sequencing comparing primary hTM cell cultures to hTM tissue may provide important insights on cellular identity and translatability, as such an approach has not been reported before. In this study, we assembled a total of 14 primary hTM *in vitro* samples across passages 1–4, including 4 samples from individuals diagnosed with glaucoma. This dataset offers a comprehensive transcriptomic resource of primary hTM *in vitro* scRNA-seq data to study global changes in gene expression in comparison to cells in tissue *in situ*. We have performed extensive preprocessing and quality control, allowing the research community to access and utilize this public resource.

## Background & Summary

Primary open angle glaucoma (POAG) is a devastating ocular disorder resulting in irreversible vision loss. While the precise etiology and progression of POAG are complex, reducing intraocular pressure (IOP) is the only modifiable risk factor for managing visual field loss. The trabecular meshwork (TM) in the anterior segment of the eye is a major site of egress for aqueous humor^1^. Dysfunction of the TM, contributed by tissue resident cells and the extracellular matrix (ECM), is thought to result in increased resistance to aqueous drainage and ocular hypertension (OHT)^2,3^. The TM is thought to be anatomically heterogeneous and is comprised of 3 regions: the juxtacanalicular region (JCT) within the cribriform region, corneoscleral meshwork, and uveoscleral meshwork, of which the area around the juxtacanalicular region (JCT) of the meshwork and the inner wall cells of the Schlemm’s canal is considered the major site of resistance to outflow^4–8^. The primary intervention to reducing OHT is achieved through targeting either reduction of aqueous production (e.g. *α*-adrenergic agonists, *β*-blockers, carbonic anhydrase inhibitor), or via increasing outflow via the unconventional pathway (e.g. prostaglandins, miotic & cholinergic agents). More recently, Rho kinase inhibitors and nitric oxide donor drugs that target the conventional outflow apparatus have been approved for use.

Understanding the molecular pharmacology and mechanism of action of drugs has often relied on primary TM cells cultured *in vitro* prior to conduction of *ex vivo* or *in vivo* studies. However, translation of *in vitro* efficacy to pre-clinical and subsequently clinical outcomes remains a challenge. Differences in tissue anatomy of ocular structures, physiology, microenvironment, and species all contribute to the inability to replicate the complexities of an organism *in vitro*. The physiology and anatomy of the TM is complex with functional heterogeneity observed in the form of segmental regions of high, intermediate and low flow^9^. Segmental heterogeneity is associated with changes in extracellular matrix composition, biomechanical properties, and molecular signaling pathways in cells^10–14^. To further understand this complex tissue, single cell transcriptomic studies demonstrate heterogeneity in cell types within the conventional pathway with 12-to-19 distinct cell types identified with region-specific expression of candidate genes to define cellular identity^15,16^. In contrast, *in vitro* cell culture of primary human TM (hTM) cells originate with isolation of these cells from TM tissue dissected out of human donor anterior segment, corneal rims or whole globes. Though these primary cells are utilized for *in vitro* studies in early passages (∼2–6), there is a growing recognition among the community that cellular identity is heterogeneous, may change with time, and is variable from donor-to-donor. Furthermore, there is a considerable interest in the concept of mechanical memory of cells that may help define cellular identity and phenotypic characterizations.

**Fig. 1:**
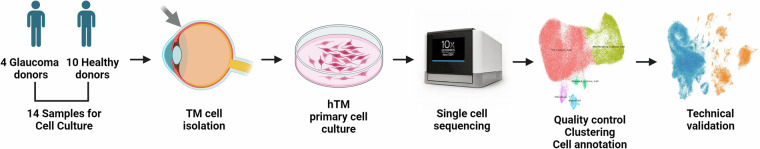
Schematic of experimental workflow.

In this study, primary hTM cells were isolated and cultured from non-glaucomatous and glaucomatous donors following tissue dissection, validated through cobblestone morphologic appearance and dexamethasone-induced myocilin, and compared with freshly dissected human TM tissue via single cell transcriptomics. Several TM cell specific marker genes were identified, e.g. Chitinase 3 Like 1 (*CHI3L1*), matrix gla protein (*MGP*), and myocilin *MYOC* albeit within different clusters. Prior comprehensive transcriptomic analysis demonstrates the aforementioned genes to be present in the TM and other ocular tissues^15–18^ affirming that the cells characterized are indeed from the appropriate tissue isolated (see Fig. 1 for workflow). When the transcriptome of these primary hTM cells were superimposed with those of TM tissue, a striking divergence in cell composition was observed. Specifically, we observe dramatically reduced cell heterogeneity and changes between transcriptomics profiles in the *in vitro* culture compared to *in vivo* tissue.

## Methods

### Isolation of donor primary hTM cells

Primary hTM cells were isolated from human donor corneal rims deemed unsuitable for transplantation, and characterized as described previously^19,20^. All donor tissues for primary cell culture were procured from Saving Sight eye bank (Kansas city, MO). All primary cell culture donor cells (Tables 1, 2) used in this study were isolated (between 2012 and 2022), validated, stored in liquid nitrogen, or used in prior studies from our lab^14,21–24^. Frozen cells were thawed with 15 mL pre-warmed media (37 °C, DMEM:F12 = 1:1, with 20% FBS, 1% Penn/Step/fungizone) and centrifuged at 300 g, 4 °C for 5 minutes. Cells were washed with media again and suspended in 0.04% BFA. Cell viability was checked with trypan blue.

**Table 1:** Donor information and sample IDs for primary cell culture samples.

Donor_ID	Sample_ID	Sample Type	Disease	Side	Passage
GTM_1672	10x3V31_GTM_1672_P3	primary cell culture	glaucoma	unknown	P3
GTM_2956	10x3V31_GTM_2956	primary cell culture	glaucoma	OS	P1
GTM_7445	10x3V31_GTM_7445	primary cell culture	glaucoma	unknown	P4
hTM_0114	10x3V31_hTM_0114_P1	primary cell culture	healthy	OS	P1/P2
hTM_11701	10x3v31_hTM_11701_P2	primary cell culture	healthy	OS	P2
hTM_11703	10x3v31_hTM_11703_P2	primary cell culture	healthy	OS	P2
hTM_2180	10x3v31_hTM_2180	primary cell culture	healthy	OD	P1
hTM_659	10x3V31_hTM_659_P1_DCR	primary cell culture	healthy	unknown	P1/P3
hTM_74M	10x3V31_hTM_74M_P2	primary cell culture	healthy	OD	P2
hTM_7987	10x3V31_hTM_7987_P2	primary cell culture	healthy	unknown	P1/P2
hTM_9355	10x3v31_hTM_9355_P1	primary cell culture	healthy	OS	P1
hTM_9632	10x3v31_hTM_9632_P2	primary cell culture	healthy	unknown	P2
hTM_9691	10x3v31_hTM_9691_P1	primary cell culture	healthy	unknown	P1
OHSU_GTM_2019_0461	10x3V31_OHSU_GTM_2019_0461	primary cell culture	glaucoma	unknown	P3
22_0500_TM	BCM_22_0500_TM	Post-mortem tissue	healthy	unknown	NA
22_0688_TM	BCM_22_0688_TM	Post-mortem tissue	healthy	unknown	NA
22_0769_TM	BCM_22_0769_TM	Post-mortem tissue	healthy	unknown	NA

**Table 2:** Donor information for post-mortem TM samples.

Donor	Age	Gender	Postmortem Time (Hr)	Ethnicity
BCM_22_0500	64	M	8	white
BCM_22_0688	68	M	20	white
BCM_22_0769	11	M	10	white

### Donor tissues

TM tissue for scRNAseq was dissected from donor anterior segments/whole globes obtained from Lions Eye Bank (Baylor College of Medicine, Houston, TX) within 4–6 hours post-mortem. Dissection was performed in accordance with consensus guidelines^19^.

### Single-cell RNA sequencing

Resuspended single cells were loaded on a 10X Chromium controller for obtaining single cell Gel Beads-In-Emulsions. scRNA-seq libraries were generated using 10X Chromium Single Cell 3′ reagent kits v3.1 (10X Genomics) following the manufacturer’s recommendations (https://www.10xgenomics.com). Sequencing was performed on Illumina Novaseq. 6000 (http://www.illumina.com) at the Single Cell Genomics Core at Baylor College of Medicine.

### Meta-analysis of scRNA-seq datasets

#### Preprocessing of scRNA-seq Datasets

Raw sequencing reads were processed using the CellRanger v6.1.2 (10X Genomics) pipeline, against the hg38 reference genome (https://cf.10xgenomics.com/supp/cell-exp/refdata-gex-GRCh38-2020-A.tar.gz). Then, quality control was performed separately for each sample through a quality control pipeline (https://github.com/lijinbio/cellqc)^25,26^. Real cells were filtered by dropkick v1.2.8^27^ and ambient RNA was removed using SoupX v1.6.2^28^. Doublets were detected and removed using DoubletFinder^29^. After merging all samples, cells were further filtered manually (features >300, transcript (UMI) counts >500, mitochondrial percentage <5%) (Table 3).

**Table 3:** Cell count after each QC step.

SampleID	Dropkick	SoupX	Common	DoubletRemoval	Final Filtering
10x3V31_GTM_1672_P3	10380	11699	8791	8197	6910
10x3V31_GTM_2956	6400	8611	5007	4814	3257
10x3V31_GTM_7445	10280	9139	8286	7758	6943
10x3V31_hTM_0114_P1	8473	9162	6505	6179	5682
10x3v31_hTM_11701_P2	8285	11929	7391	6971	5025
10x3v31_hTM_11703_P2	7878	18391	7360	6943	6012
10x3v31_hTM_2180	16985	19081	16615	14491	13340
10x3V31_hTM_659_P1_DCR	7312	9491	7023	6644	991
10x3V31_hTM_74M_P2	12131	12384	9591	8883	7839
10x3V31_hTM_7987_P2	4945	7098	4101	3972	3709
10x3v31_hTM_9355_P1	9347	11560	7951	7465	6071
10x3v31_hTM_9632_P2	7113	8576	6563	6232	3547
10x3v31_hTM_9691_P1	7328	8545	6687	6343	4678
10x3V31_OHSU_GTM_2019_0461	12754	13404	9827	9084	8539

*Common cells between Dropkick and Cellranger outputs were used for the next step of QC.

#### Data integration and clustering

The raw counts from each sample were merged, and the scVI model (n_layers = 2, n_latent = 30) from scvi-tools v0.19.5^30^ was used to infer a latent space of 30 dimensions from the raw counts of the top 2000 highly variable genes (HVGs) calculated by scanpy v1.9.1^31^. Sample ID was provided as a batch covariate for the scVI model. The latent space was further reduced using UMAP (min_dist = 0.3), and leiden clustering (resolution = 0.5) was performed on the scVI latent space, using a k-nearest neighbor graph (neighbors = 20).

#### Cell clustering and cell type annotation

Cell cycle effects were removed using Seurat^32^. The clustering resolution was validated using sccaf^33^. Initial cell type annotation was performed using scPred^34^ with a reference dataset generated from *in vivo* tissue samples from our lab. Clusters were re-annotated using known cell type marker genes and cell types were assigned to each cluster using scanpy^31^ with specific markers from previous publications^15,16^. The detailed markers for each cell type are listed in Table 4. Cell proportion plots were generated using dittoseq^35^.

**Table 4:** Highly expressed genes of individual cell type from previous publications.

Cell Type	Highly-Expressed Genes	
Macrophage	C1OB, MSR1, MARCO	
T/NK	NKG7, KLRB1, CD3D	
Epithelium	KRT5, SFN, AOP5	
Melanocyte	MLANA, PMEL, TYRP1	
Schwann-like	L1CAM, NGFR, SOX2	
Myelinating Schwann	NGFR, SOX2, PLP1, MPZ	
TM1 (fibroblast-like)	DCN, PDGFRA, TAGLN	
TM2 (myofibroblast-like)	DCN*, PDGFRA, TAGLN, ACTA2, RGS5	
Smooth Muscle	TAGLN, ACTA2, DES, MYH11, RGS5	
Pericyte	DCN, TAGLN, ACTA2, RGS5, PDGFRB, FLT1	
Vascular Endo	RGS5, VWF, FLT1, DLL4, KDR, PECAM1	
Lymphatic-like Endo	KDR, PECAM1, MMRN1, FLT4, PROX1	Regneron
K-Epi	PAX6	Sanes
Schwalbe Line	CA3, MGARP, ADRB2, SLC2A1, IGFBP2	
Fibroblast	COL14A1, ADH1B, FBLN2, COL1A2, COL6A1, TNXB, TIMP2, FBLN1, DCN, PCOLCE	
CC	PLVAP, FLT4, PROX1, ACKR1, AQP1, SELE, MMRN1, PECAM1, VWF	
Vascular Endothelium	ALPL, SLC2A1, CLDN5	
SC	TFF3, PLAT, FN1, POSTN, CCL21, CDH5	
JCT	NGPTL7, PDPN, CEMIP, MYOC, CYTL1, CHI3L1, NEB, RSPO4, FMOD, NELL2	
BeamA	BMP5, MGP, RARRES1, FABP4	
BeamB	TMEFF2, PPP1R1B, BAMBI	
CM	CHRM3, DES, CNN1, MYH11, MYLK, ATP2A1	
Pericyte	NDUFA4L2, FABP4	
Neuron	CHRNA3,CALB2,UCHL1,SCG2,GAP43	
Melanocyte	MLANA,PMEL,MITF,TRPM1,TYR	
Myelinating Schwann	MBP,MPZ,PLP1	
Non-Myelinating Schwann	LGI4,CDH19	
BCell	CD27,CD79A,IGHM,MZB1	
Macrophage	LYVE1,CD68,CXCL8,IL1B,TREM2	
Mast Cell	RGS13,KIT,CD2	
NK/T	CD3D,IL7R,TRAC,NGK7	

#### Differentially expressed gene analysis

To identify genes that are differentially expressed between cell types, genes specifically expressed in each cluster were identified and the top 5 genes expressed in each cluster were ranked using Seurat^32^.

#### Data integration with *in vivo* dataset

Cell type of three samples of *in vivo* hTM cells was determined with scPred individually and then an integrated object of only *in vivo* data was compared with the same known cell type marker genes to determine validity of the *in vivo* samples. Data integration with the tissue culture data was then performed following the same parameters and protocol as above. Then, the combined tissue + culture object was once again compared with the known cell type marker genes and a disparity in gene expression was observed.

## Data Records

Raw reads of all samples and processed data files including integrated data were deposited in the Gene Expression Omnibus (GEO, https://ncbi.nlm.nih.gov/geo) of the National Center for Biotechnology Information (NCBI) as FASTQ files with accession number GSE263230^36^.

## Technical Validation

**Fig. 2:**
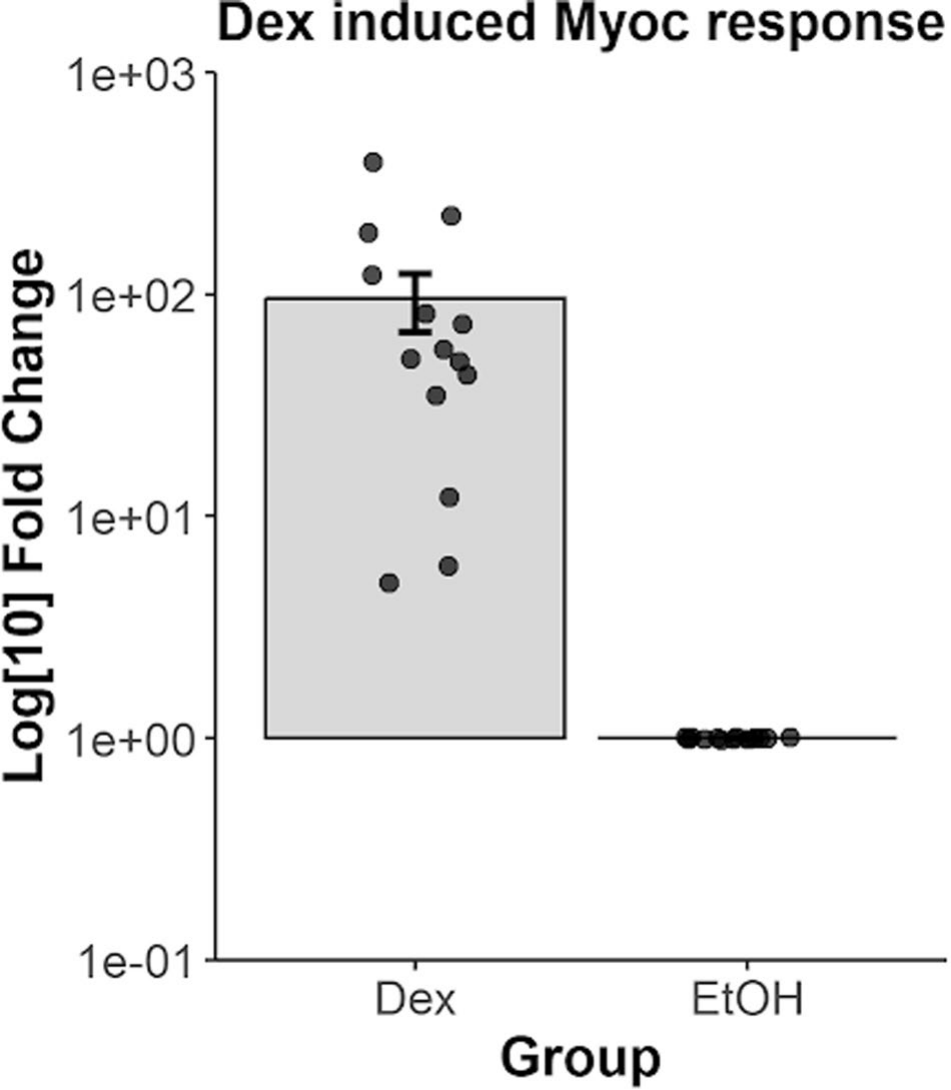
Primary hTM cells used in study demonstrated elevated *MYOC* expression in response to 100 nM dexamethasone treatment for 3 days. Data are from n = 14 donors represented as a bar graph, mean ± standard error in mean. ***p < 0.0041, t-test.

**Fig. 3:**
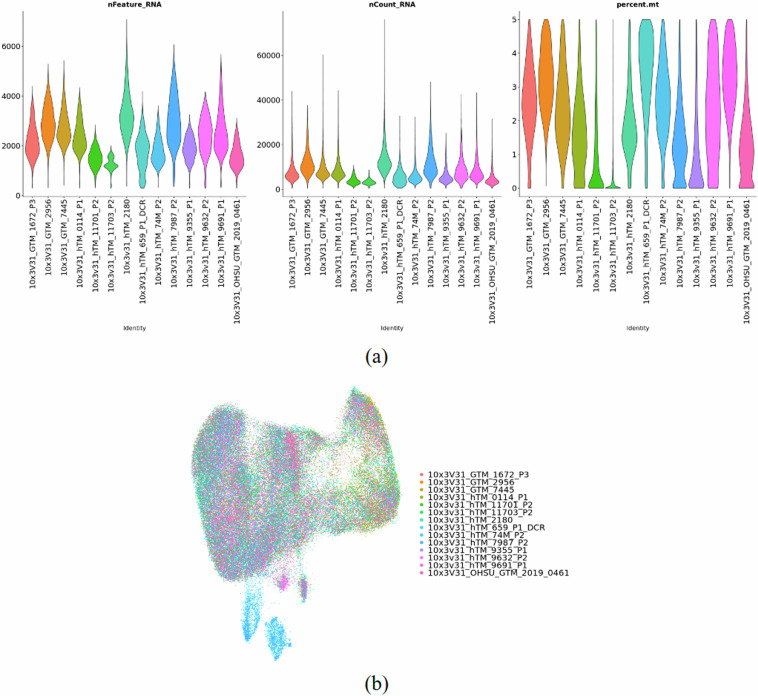
Quality Validation and Characterization of Cells. (**a**) Violin plots showing number of features, number of counts, and mitochondria percentage by sample. (**b**) The distribution of cells by sample.

Prior to sequencing experiments, hTM cells were simultaneously validated by documenting *Myoc* mRNA expression in response to 100 nM dexamethasone treatment for 3 days (Fig. 2). Quality control was performed on each sample independently with an average of 6000 cells profiled, yielding a total of about 82k cells (Table 3). There are an average of 2300 gene counts and 8400 UMI counts over the 14 samples (Fig. 3(a)). After integration, we affirmed that batch effects were not significant (Fig. 3(b)).

**Fig. 4:**
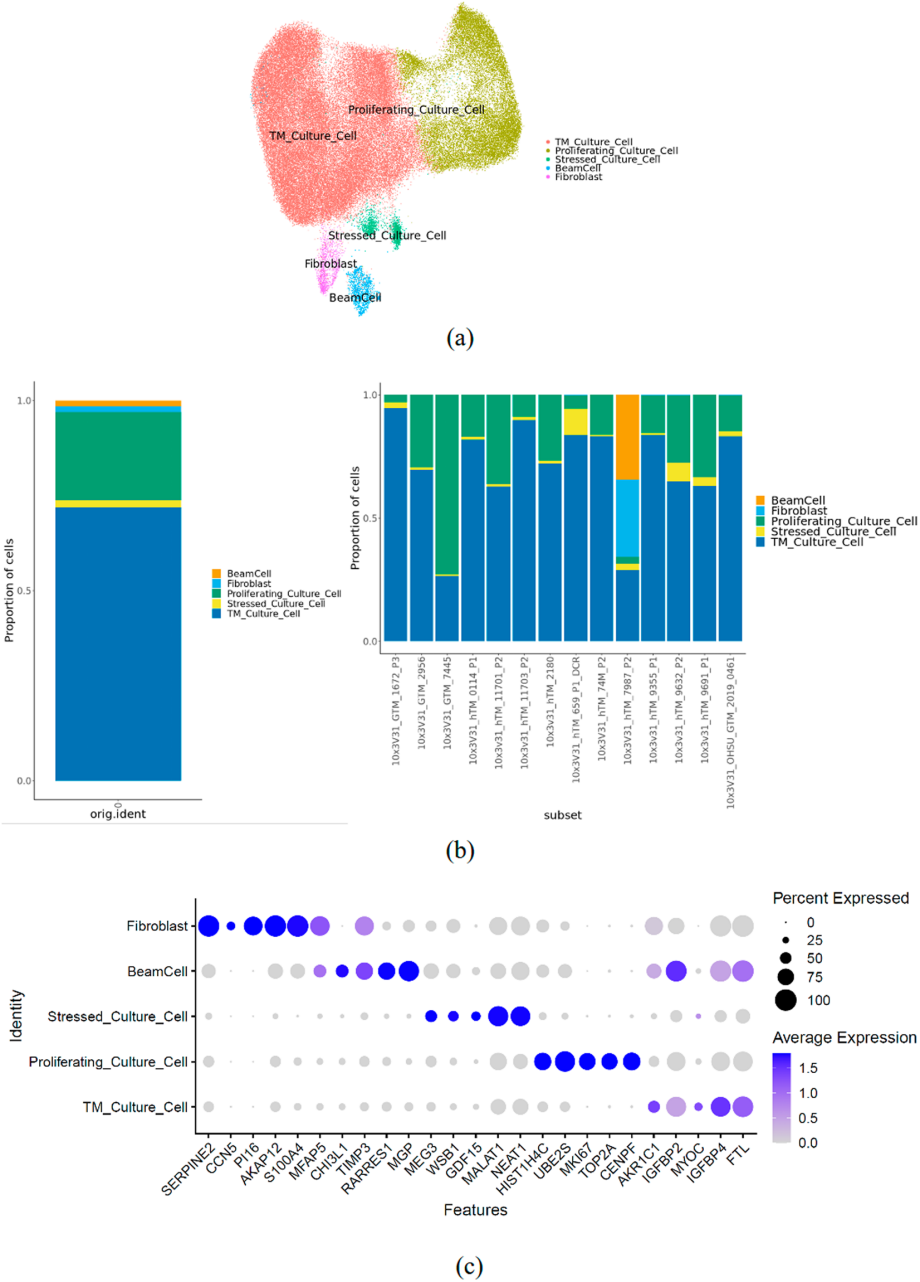
Quality Validation and Characterization of Cells. (**a**) Umap of integrated object with cell type annotations. (**b**) Cell proportion bar plot. (**c**) Dotplot with top 5 highly expressed genes.

By performing clustering analysis of all the cells, a total of 5 clusters were obtained. Of the 5 clusters identified, 3 clusters (TM_Culture_Cell, Proliferating_Culture_Cell, and Stressed_Culture_Cell) are shared by all donors (Fig. 4(a)). The other two clusters are only present in the hTM_7987 sample (Fig. 4(b)). This suggests that the population of hTM cells in culture is non-uniform and exhibits some heterogeneity. Several TM cell specific marker genes were identified; e.g. CHI3L1, MGP, and MYOC within different clusters. While MYOC was primarily expressed in the TM_Culture_Cell cluster, CHI3L1 and MGP were abundant in the BeamCell cluster. When overlaid with cell-specific markers previously reported, PDPN, CEMIP and CHI3L1 were abundant in the BeamCell cluster, while MYOC remained enriched in the TM_Culture_Cell cluster. Matrix proteins, collagens 1/6, FBLN, FN, POSTN, and DCN were enriched in clusters BeamCell and Fibroblast, while PCOLCE was enriched in clusters TM_Culture_Cell and Proliferating_Culture_Cell (Fig. 4(c)). Prior comprehensive transcriptomic analysis demonstrates the aforementioned genes to be present in the TM and other ocular tissues^15–18^ affirming that the cells characterized are indeed from the appropriate tissue isolated. Further, pairwise correlation analysis of psuedo-bulk transcriptomic profile expression of cells was made comparing our study with the previous study^16^ (Supplementary Fig. 1). Data demonstrated significant correlation, indicating good concordance in cell identities and characterization between both studies.

**Fig. 5:**
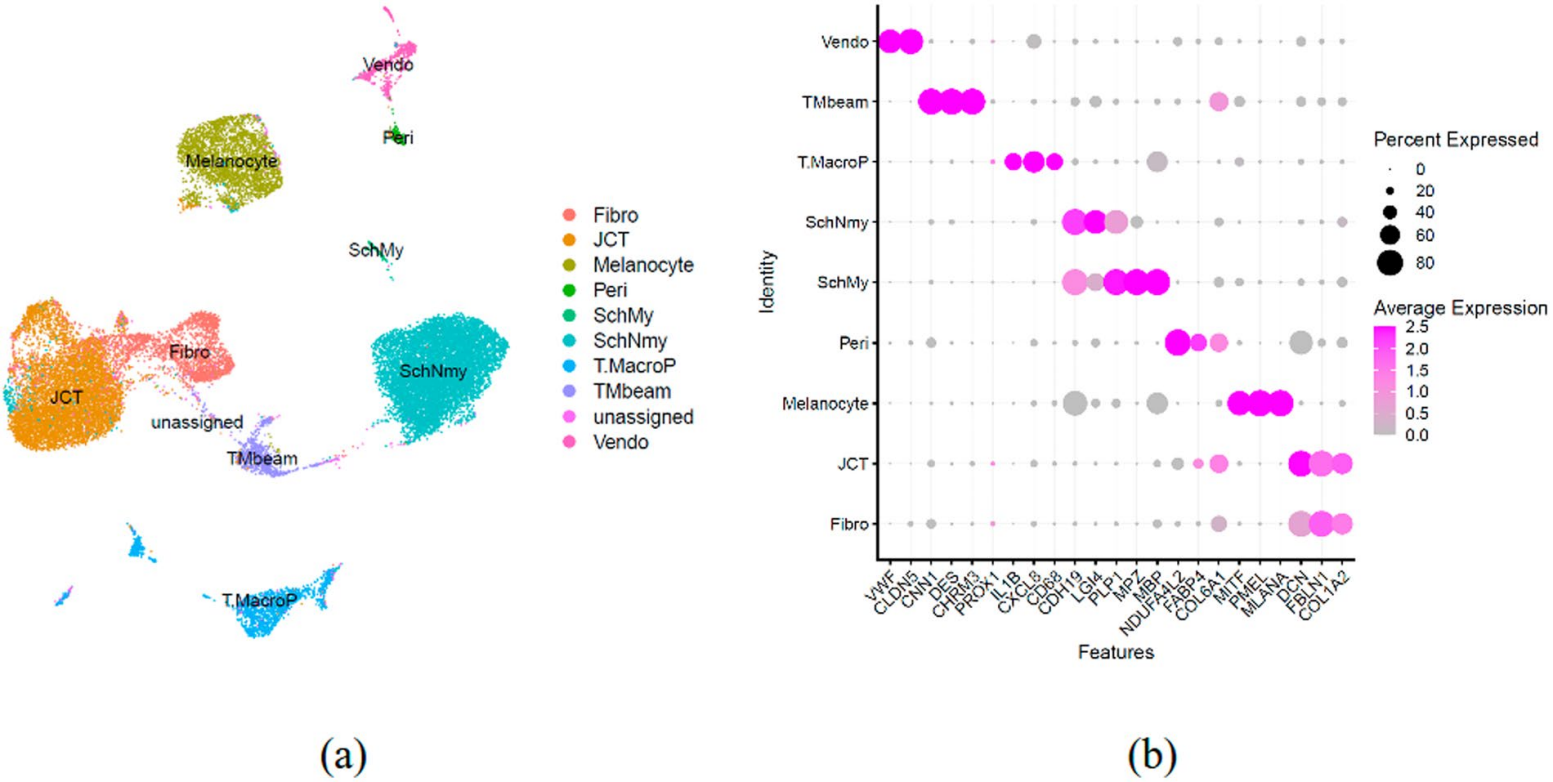
Single cell transcriptome of TM tissues for quality validation. (**a**) UMAP of *in vivo* hTM cells with cell type annotation by Seurat. (**b**) Dot Plot showing marker gene expression by cluster.

With the tissue data, we used the same QC pipeline with the same parameters for prepossessing. In the tissue samples alone, there are an average of 8100 cells profiled, yielding a total of about 24k cells with an average of 2300 gene counts and 6400 UMI counts. We assigned cell types using scPred to yield 9 defined cell types with a few unassigned cells (Fig. 5(a)). We validated the clustering and cell type assignment with previously identified marker genes^15–18^ and confirmed that the data were suitable for further analysis.

## Usage Notes

**Fig. 6:**
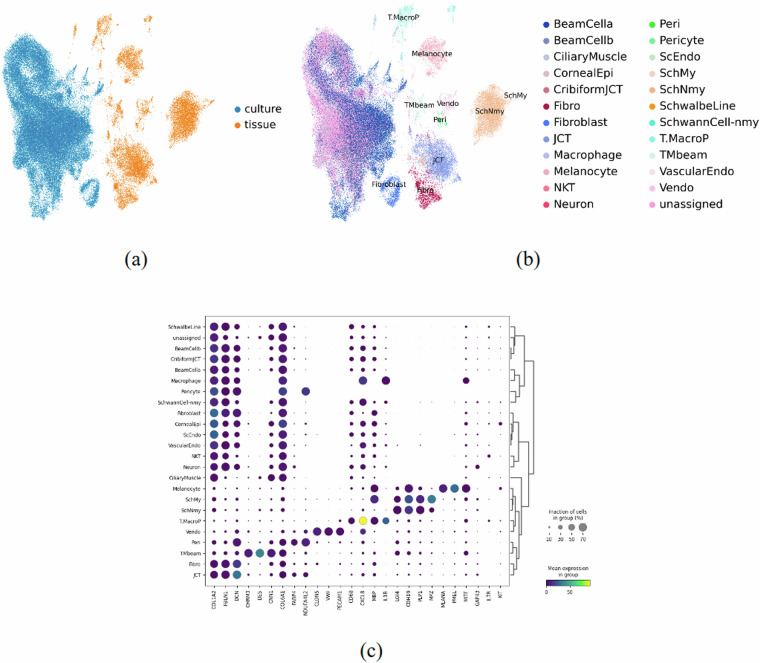
Data integration for comparison of *in vivo* and *in vitro* data. (**a**) UMAP by sample type with separation of clusters between primary cell culture and post-mortem tissue. (**b**) UMAP by cell type with separation of clusters between primary cell culture and post-mortem tissue. (**c**) Dot Plot showing marker gene expression by cluster between both sample types.

Our dataset will be useful for a variety of studies pertaining to understanding the identity of primary hTM cells, including studies to translate pre-clinical *in vitro* models to *in vivo* models mimicking disease, relevant biophysical and biochemical cues mimicking the native cellular microenvironment, and choice and type of *in vitro* models utilized for investigations. Here we provide a comparison between our dataset and tissue data generated by our lab as a usage example:

When the transcriptome of these primary hTM cells were superimposed with those of TM tissue, a striking divergence in cell identity was observed. Specifically, reduced cell heterogeneity and dramatic changes of transcriptomics profiles in the *in vitro* culture compared to *in vivo* tissue (Fig. 6). Importantly, we note that a comparison of primary hTM cells cultured from glaucomatous and non-glaucomatous donors demonstrated no significant differences in cell type markers or clusters identified (Supplementary Fig. 2,3). Furthermore, regardless of disease state, primary hTM cells differ in their transcriptome profile compared with human tissue. We also note that primarily the tissues and cells were isolated from donors with similar reported ancestry (white). As such, differences observed between cultured cells and tissues is unlikely due to race. It is important to note that the primary hTM cells characterized in this study were previously frozen down in liquid N2 and are from early passages (up to 2, or as mentioned in Table 1). It is unclear why and at what stage these putative markers may be lost in culture. We are also uncertain whether, at any point during culture, these cells may have de-differentiated from a specific population of proliferating cells, as the heterogeneity observed *in vitro* is significantly reduced compared to that in the tissue. Nevertheless, while it is currently out of scope, we anticipate that future investigations will perform secondary validation efforts comparing cell specific marker expression in both primary TM cells in culture and tissues.

Our group and others have consistently reported that biophysical and biochemical cues from the cell culture microenvironment (topography, stiffness, ECM coating, stretch, 2D vs 3D) have profound impact on transcriptomic, proteomic, signaling pathways, and response to drugs *in vitro*^37–56^. Thus it is feasible to infer that while mechanical cues may drive hTM cell function as a function of substrate properties, the initial culture conditions in which these cells were first isolated and expanded may have also profound impact on selection of cells for propagation, proliferation, and (de-)differentiation. That mechanical memory and plasticity exists in maintaining cell identity through epigenetic regulation has been previously postulated^57–65^. However, whether such a phenomenon exists in primary hTM cells remains to be further determined. Emerging evidence suggests chromatin remodeling implicating significant changes to cellular and epigenetic plasticity may significantly alter cell fate with prolonged exposure to rigid culture environments such as tissue culture plastic^62,63,66–68^. Newer studies have also suggested that the cytoskeleton and transcriptional elements may have additional roles in cellular plasticity and memory, though the evidence for such is currently limited^69–71^. It is further interesting to note that cellular memory to external and innate stimuli is well documented for immune cells. However, whether such responses translate to non-immune cells is unclear although it is likely such mechanisms may be conserved. Whether these responses translate in the form of ECM remodeling, transcriptional & translational regulation, cytoskeletal reorganization, cell division and/or phenotypic outcomes warrants mechanistic investigation. For example, the evidence pertaining DNA methylation/cellular differentiation is shown to be dependent on cell type (e.g. stem cells vs terminally differentiated cells^72–74^) and substrate mechanical properties. The above studies were done *in vitro* and lacked a direct comparison to cells *in vivo* consequently highlighting some paucity in our understanding of these phenomena. Since all primary hTM cells are are terminally differentiated cells that are primarily cultured and expanded on rigid plastic substrates, it is only natural to infer that the process of divergence in cell identity as observed in this study likely starts immediately after initial isolation. A systematic study is critically needed to confirm this, and this may subsequently allow for the development of appropriate culture conditions and microenvironments which better maintain primary TM cell identity. Since a mechanistic approach was not taken in this study to ascertain why, how and when the divergence in cell identity comparing primary culture to tissues occurs, we point the audience to relatively recent manuscripts/editorials that attempt to draw highlight to this^75–77^. With increasing efforts in the use of organoids, microphysiological systems, bioprinting etc, integration of physiological, chemical, mechanical, spatial profiling, and multi-cell complexities may help develop better models for studying outflow homeostasis and dysfunction^75,78,79^.

All raw RNA sequencing data are stored in FASTQ files, and processed.h5ad files are also available for use.

## Supplementary information

Supplementary Information

## Data Availability

The source code, including code to generate all figures, has been uploaded to GitHub: https://github.com/RCHENLAB/TM_culture_manuscript.
