# Supplementary material for: Divergence in cellular markers observed in single-cell transcriptomics datasets between cultured primary trabecular meshwork cells and tissues
Source: Sci Data. 2025 Feb 14;12:264. doi: 10.1038/s41597-025-04528-5 (PMC11829053; doi:10.1038/s41597-025-04528-5)
Supplement: Supplementary file 1 — Supplementary Information [file 41597_2025_4528_MOESM1_ESM.pdf]

Supplementary figure 1. comparing TM tissue data sets between Chen and Sanes

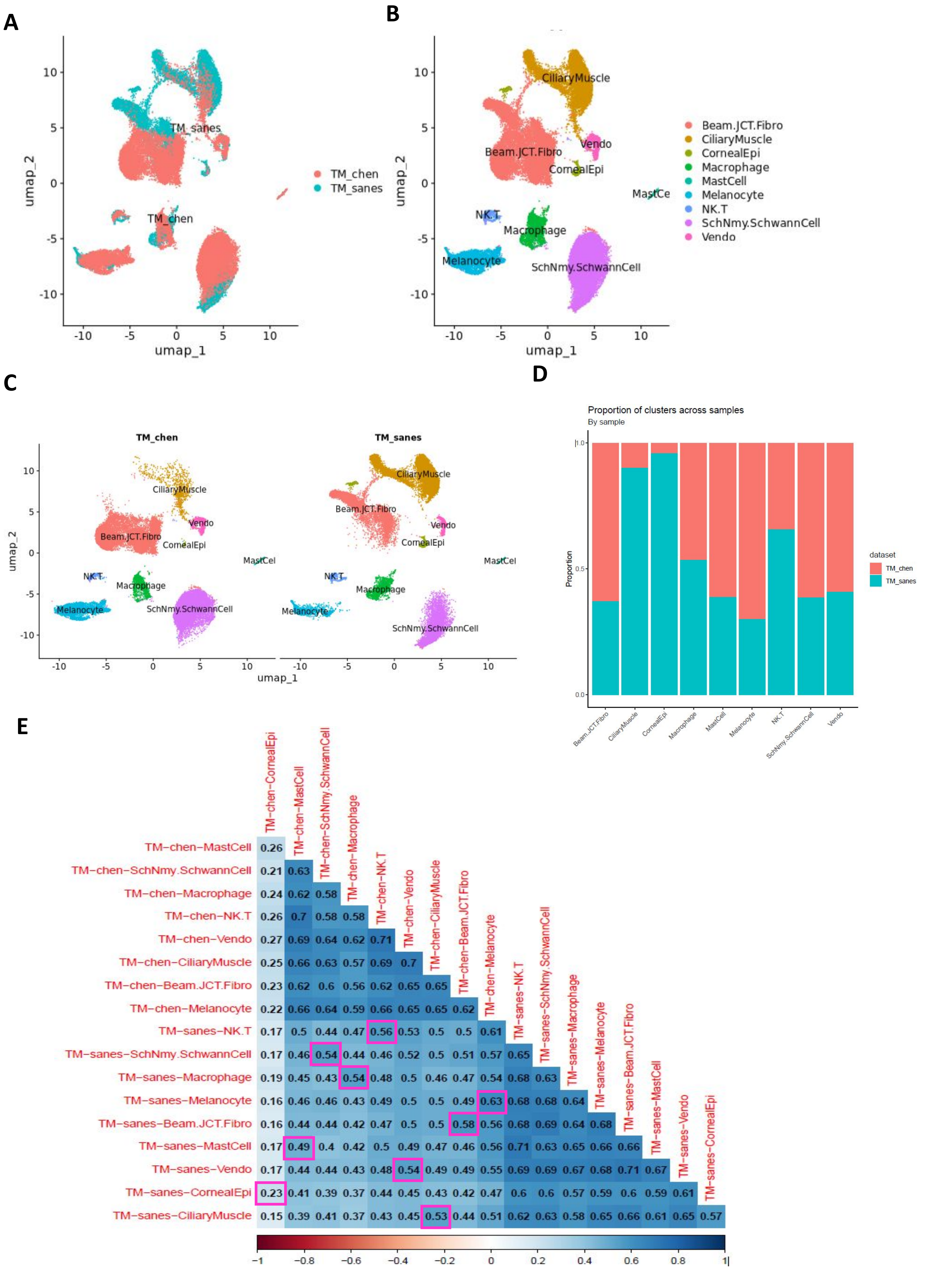

$p < 0.001$

Supplementary figure 2. comparing norm(n=10) vs glaucoma(n=4) sample data

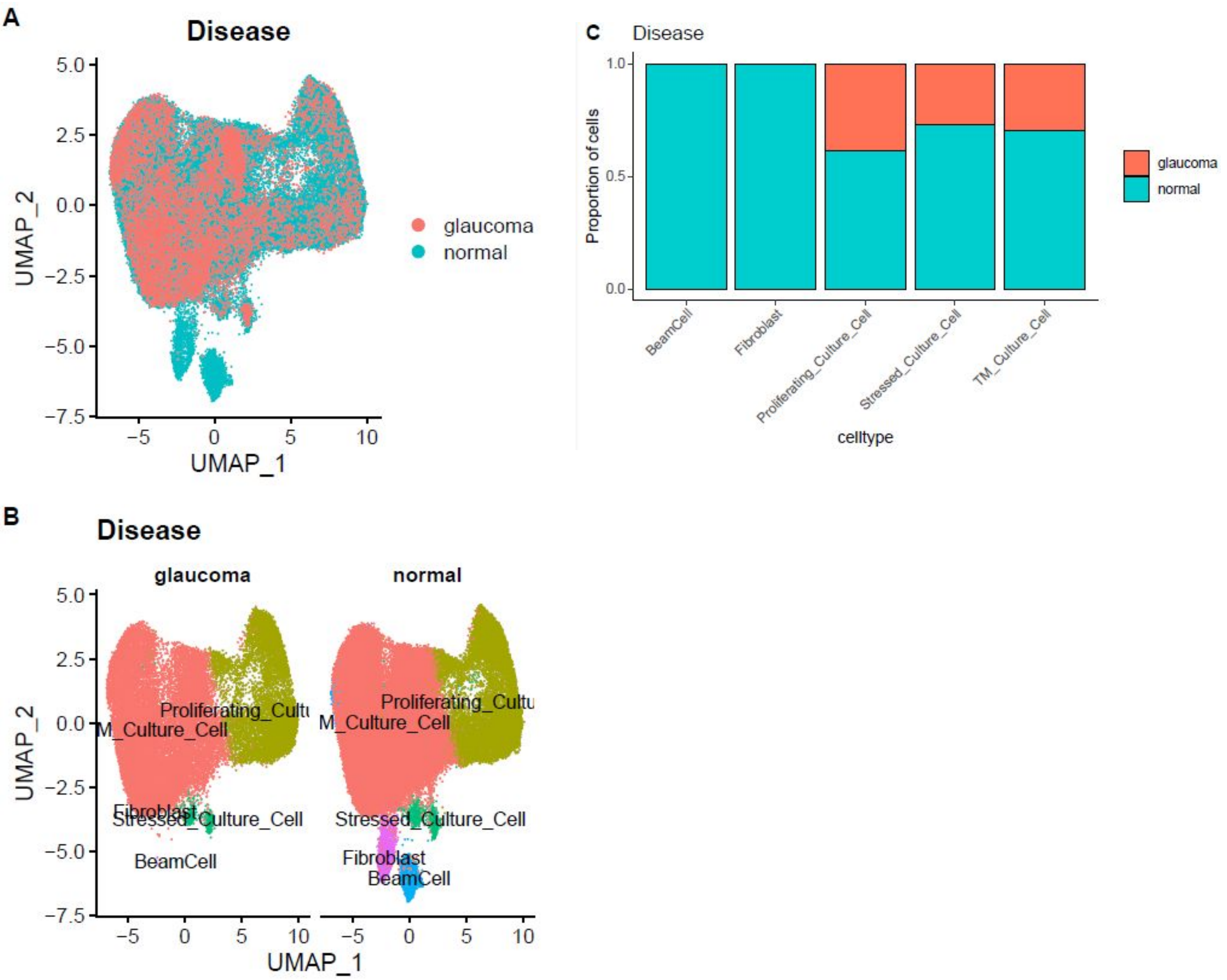

Supplementary table1. Number of cells assigned to individual cell clusters

	Normal cells		Glaucomatous cells		
**celltype**	**cells**	**Proportion (%)**	**cells**	**Proportion (%)**	**Total**
TM_Culture_Cell	41591	73.1	17772	69.3	
Proliferating_Culture_Cell	11734	20.6	7457	29.1	
Stressed_Culture_Cell	1118	2	415	1.6	
BeamCell	1273	2.2	1	0	
Fibroblast	1178	2.1	4	0	
**Total**	56894	100	25649	100	82543

Supplementary figure3. Scatter plots for correlation of highly variable genes (HVGs, n=2000) between normal and glaucoma

A. TM culture cell

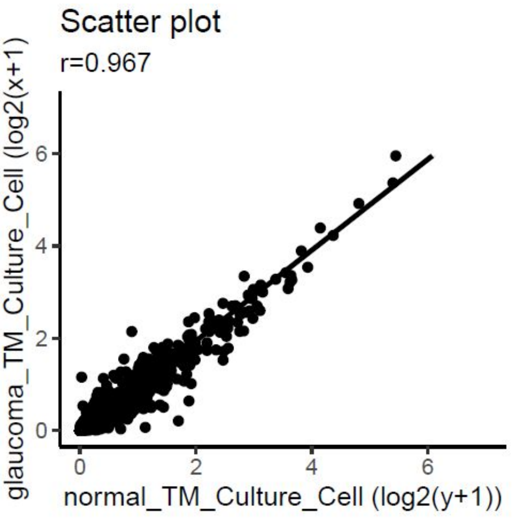

B. Proliferating Culture Cells

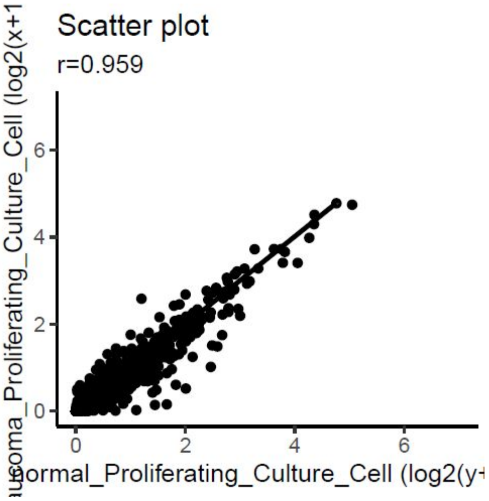

C. Stressed culture cell

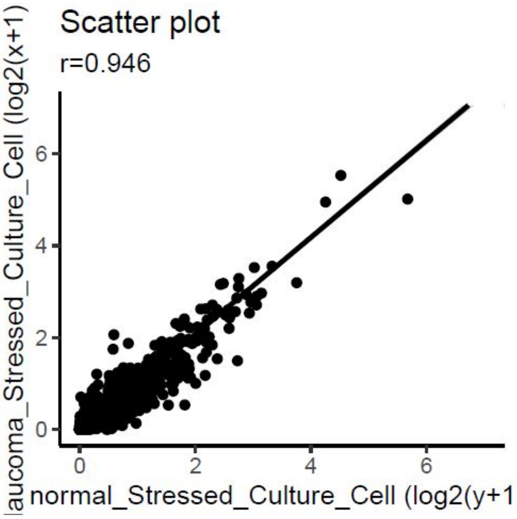

Supplementary figure 4. Sub-group analysis: superimpose normal_cells to normal TM tissue data

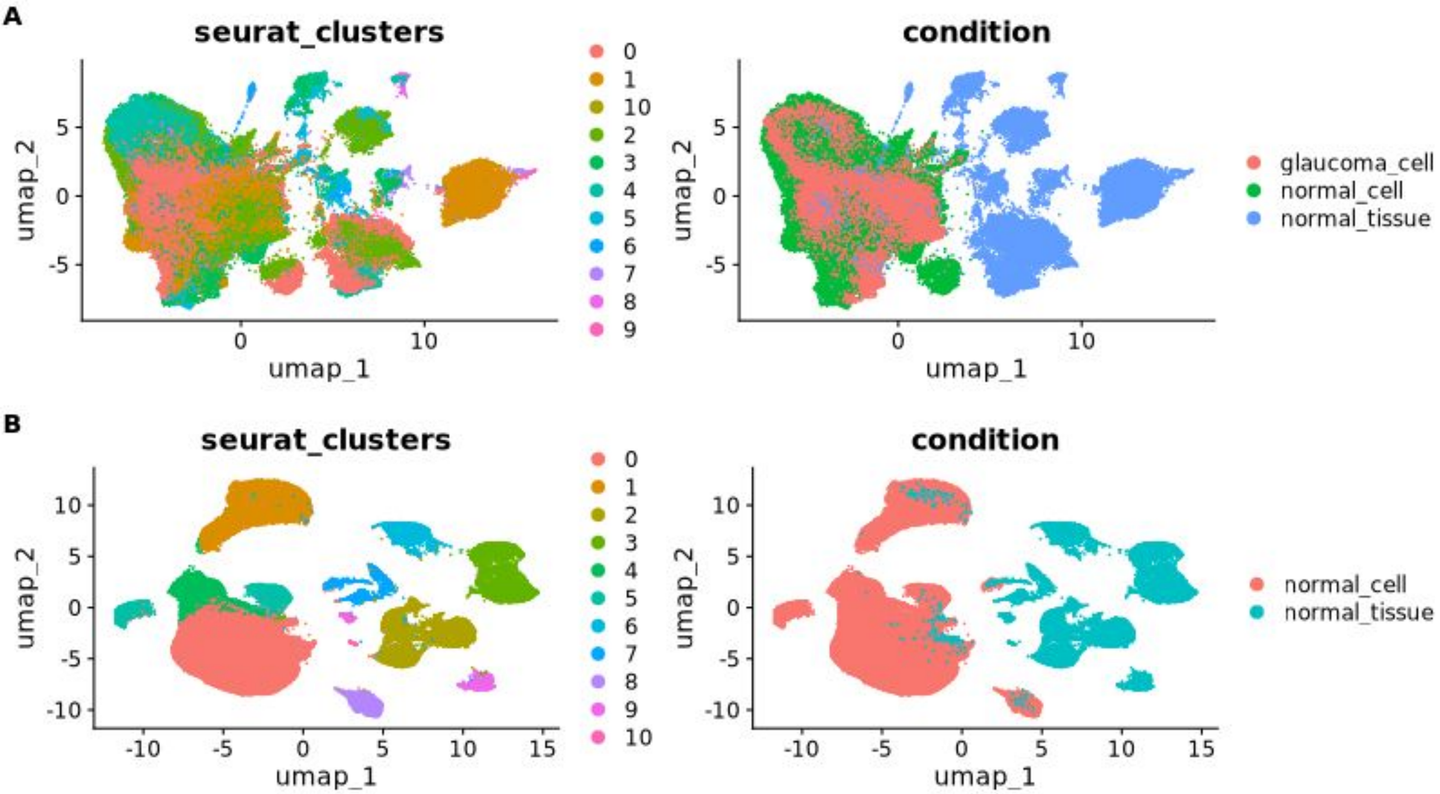

A. Data integration and clustering for 3 groups: glaucomatous cells, normal cells, and normal tissues from Chen lab. B. Data integration and clustering for 2 groups: normal cells, and normal tissues.

Supplementary figure 5. Further sub-clustering of hTM cell data and assessing cell types using published known cell markers

A. rs=0.1

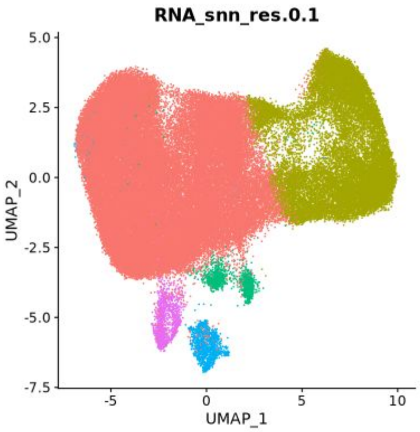

Known cell markers from Sanes group in Table4*

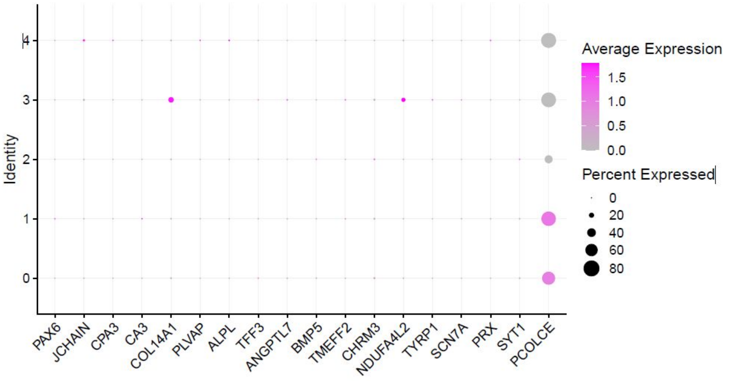

B. rs=0.3

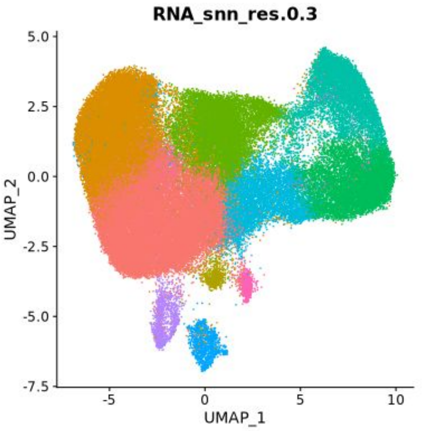

Known cell markers from Sanes group in Table4*

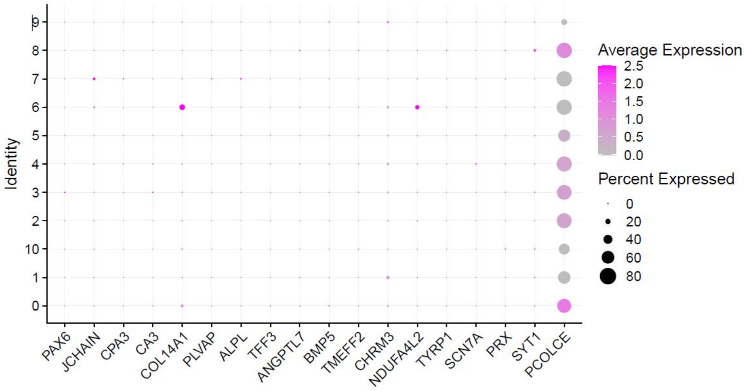

C. rs=0.5

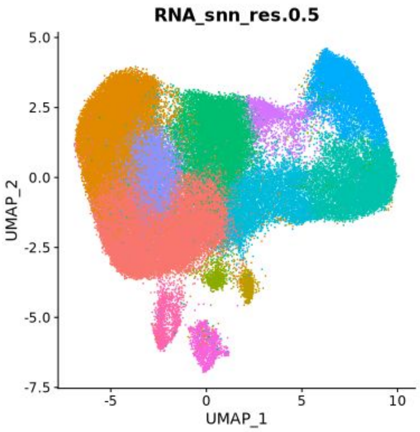

Known cell markers from Sanes group in Table4*

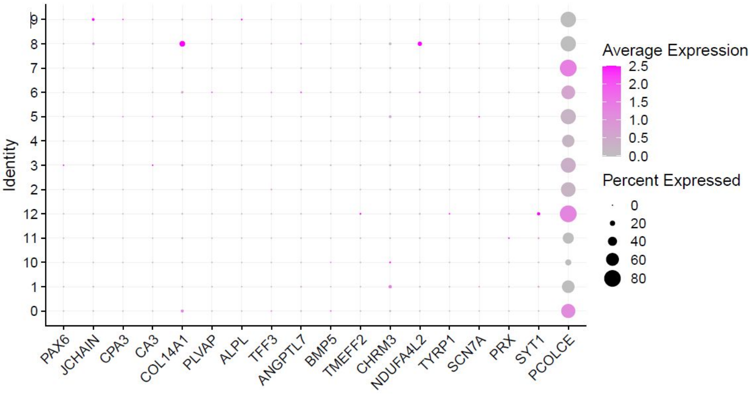

* Known cell markers: To assess the cell type reported cell markers used from publication (Cell Atlas of Human Trabecular Meshwork and Aqueous Outflow Structures, Zyl et al., 2000).
